# The immediate and long-term effects of prenatal opioid exposure

**DOI:** 10.3389/fped.2022.1039055

**Published:** 2022-11-07

**Authors:** Elizabeth Yen, Jonathan M. Davis

**Affiliations:** ^1^ Department of Pediatrics, Tufts Medical Center, Boston, MA, United States; ^2^ Mother Infant Research Institute, Tufts Medical Center, Boston, MA, United States; ^3^ Tufts Clinical and Translational Science Institute, Boston, MA, United States

**Keywords:** neonatal abstinence syndrome, short-term effects, long-term outcomes, nutrition, growth trajectory, brain development, ophthalmologic disorders, physical therapy

## Abstract

The opioid epidemic has adversely affected neonates and children, yet the mechanisms by which it impacts this population are not well understood. Not only does prenatal opioid exposure result in short-term consequences shortly after birth, it also creates long-term sequelae that may predispose these children to physical, emotional, psychiatric, cognitive, and socioeconomic problems in the future. This article provides a scoping overview of the long-term effects of antenatal opioid exposure on neonates and children as well as quality improvement and research efforts to understand and mitigate this major public health concern.

## Introduction

Between 1999 and 2014, the number of pregnant women with opioid use disorder (OUD) increased from 1.5 to 6.5 cases per 1,000 hospital births (1). This led to a steep increase in the number of neonates with Neonatal Abstinence Syndrome (NAS) from 1.2 to 8.0 per 1,000 hospital births, with some areas reaching 20.0 per 1,000 hospital births (2, 3). A diagnosis of NAS is based on a variety of systems that evaluate the presence and severity of withdrawal (4–11). Non-pharmacologic approaches remain the primary focus of NAS management followed the initiation of pharmacotherapy if signs are still significant. This review will discuss the definition of NAS, pharmacotherapy of NAS, longer-term neurodevelopmental outcomes, and new initiatives to monitor and potentially mitigate longer-term complications.

## The definition of NAS

With standardization of medication-assisted treatment (MAT), many pregnant women are receiving methadone, buprenorphine, and buprenorphine/naloxone. Consequently, neonatal opioid withdrawal syndrome (NOWS) is used to characterize signs of withdrawal result specifically from maternal opioid use. However, due to frequent polysubstance use during pregnancy, most clinicians continue to use the term NAS instead of NOWS.

While a diagnosis of NAS is made frequently, there is no consensus on the precise criteria. Some apply the diagnosis to: (1) all infants with a history of maternal OUD during pregnancy; (2) those with signs of withdrawal based on systems of assessment; and 3) the need for pharmacotherapy when non-pharmacologic measures are insufficient. Such variation in the definition of NAS can impact diagnostic coding, reimbursement, bedside management, research, and public health/policy (12).

To address this critical gap in terminology and the definition of NAS, a recent effort led by the US Department of Health and Human Services involved researchers, clinicians, and policy experts who proposed a simplified definition of NAS. The consensus recommendations included two key elements: (1) *in utero* exposure to opioids (with or without other substances), and (2) the presence of 2 of 5 of the most common clinical signs of NAS, i.e., high-pitched/excessive cry, poor sleep, hypertonia, tremors, and gastrointestinal issues. This *clinical definition* was intended to promote standardization of bedside management of these neonates, enhance research efforts, and promote public policy. The goal is to support the mother-infant dyad and provide services to help families impacted by the opioid epidemic. The authors acknowledged the unintended consequences of this enhanced definition and proposed foundational ethical principles while calling for the need to further validate the definition (12).

## Effects of polysubstance use on the severity of NAS

Women with OUD experience other mental health issues and the need for psychotropic medications (13, 14). Infants exposed to maternal opioids were more likely to require pharmacotherapy when co-exposed to benzodiazepines (15), tobacco (16), selective serotonin reuptake inhibitors (16–18), gabapentin (19), marijuana (20), or cocaine (21). The use of psychotropic medications in addition to prescription opioids increased the severity of NAS by two-fold compared to the use of prescription opioids alone (22). The absolute risk for severe NAS (need for pharmacologic treatment) was highest in infants co-exposed to opioids and gabapentin. Conversely, some studies showed that the risk of NAS was not affected by other psychotropic medications (23–25). It is unclear if drug-drug interactions or other factors (e.g., socioeconomic, maternal stressors, other medical or psychiatric disorders) contribute to the severity of withdrawal in infants with polysubstance exposure. There is very limited long-term data regarding these multiple exposures and comprehensive studies (adjusting for multiple confounders) are urgently needed and are being evaluated in several National Institutes of Health (NIH) supported Helping End Addiction Long Term (HEAL) studies.

## Presentation and management of NAS

Due to the continuous transplacental flow of opioids from the mother to the fetus, birth involves a sudden termination of supply and development of NAS. The *μ*-opioid receptors are ubiquitously present in the central nervous, peripheral nervous, and gastrointestinal systems. Opioid binding to these receptors inhibits adenyl cyclase, which further inhibits cyclic adenosine monophosphate (cAMP) production and downstream release of neurotransmitters (26). Cessation of opioids activates adenyl cyclase and disrupts the central, peripheral, and autonomic nervous systems that ultimately results in NAS. The onset of NAS can occur 24 h to several days after birth, depending on the half-life of the maternal opioid and other concurrent substance use.

First-line management of NAS is non-pharmacologic measures. Neonatal morphine solution is the most common opioid-replacement agent used in the US followed by methadone and buprenorphine. Non-opioid or adjunct agents include phenobarbital, clonidine, and gabapentin. Pharmacotherapy alleviates signs of withdrawal and optimizes short term physical, physiologic, and psychological functioning. A comprehensive review on the pharmacotherapy of NAS was recently published (27).

## Ongoing management and long-term effects of NAS

### Breastfeeding and use of breastmilk

Research demonstrates the benefits of breastfeeding in mother-infant dyads, especially pregnant mothers receiving MAT and not using illicit drugs (28–30). Although limited by small sample sizes, breastmilk analyses have shown that the concentrations of buprenorphine and methadone are low and pose minimal risks to neonates (31, 32). There are clear benefits of breastfeeding including less severe withdrawal, less need for pharmacotherapy, and shorter length of hospital stay (33, 34). The American Academy of Pediatrics (AAP) has recommended breastfeeding based on long-term benefits such as lower risk of type II diabetes, hypertension, and cancer in mothers and lower respiratory tract infections, diarrhea, otitis media, sudden infant death syndrome, asthma, and obesity in infants and children (28).

### Physical therapy

In response to the opioid epidemic, the American Physical Therapy Association has advocated for safer alternatives to pharmacologic management of pain (35). The Association promoted a non-pharmacologic approach to alleviate pain and treat NAS through its “#ChoosePT” campaign (36). Neonatal physical therapists can recognize different clinical manifestations of withdrawal from various pharmacologic agents. Such early recognition is crucial in allowing the physical therapist to help alleviate the signs of withdrawal. Physical therapists develop and personalize care plans based on the Synactive Theory of Development, focusing on an infant's interaction with the environment, particularly on four behavioral subsystems, i.e., 1) autonomic control, 2) muscle tone and motor control, 3) sleep-wake cycle and attention state control, and 4) sensory processing/modulation (37–39). Good communication between bedside clinical staff and physical therapists is essential in providing infants with the best care plan. Ideally infants should be calm, especially at the beginning of their waking time so that physical therapists can observe the natural sleep-wake transitions and the infant's regulation skills.

Using various standardized motor assessments such as the NICU Network Neurobehavioral Scale/NNNS (40) and Brazelton Neonatal Behavioral Assessment Scale (41), physical therapists can optimize a neonate's sensory-motor environment. Such interventions may include tactile stimulation, positioning aids to create supportive boundaries, vertical rocking, pacifier usage, and other calming strategies. Environmental controls that benefit opioid-exposed neonates include low-stimulation environments, e.g., minimal noise, dim lights, and the use of white noise. Additionally, sensory-motor integration may benefit from infant massage (41), swaddling, hydrotherapy (42), antigravity postural positioning, and slow and steady movements (43). All these interventions aim to integrate auditory, tactile, visual, and vestibular management to improve behavioral state regulation in opioid-exposed neonates.

### Nutrition and growth

Infants with prenatal opioid exposure are at risk for premature birth, lower birth weight, and a smaller head circumference (44–46). These likely result from the influence of maternal opioid/drug use on placental function and nutritional transport, which in turn may lead to fetal growth restriction (47). These neonates often experience postnatal growth issues, believed to result from a withdrawal-induced hypermetabolic state, feeding difficulties, and/or gastrointestinal disturbances (48, 49). A recent study demonstrated the molecular impact of prenatal opioid exposure on the hypothalamic and reward genes that regulate feeding behavior, indicating that *in utero* opioids can affect feeding regulation resulting in subsequent feeding difficulties and growth failure (50).

Because of the smaller size and postnatal growth failure, studies examined whether higher caloric intake could provide better nutritional support for opioid-exposed neonates. Infants randomized to 24 kilocalories per ounce (kcal/oz) formula had greater weight gain compared to those receiving standard 20 kcal/oz formula indicating that more calories are needed to provide ideal nutritional support in NAS (48). Another study showed that the high-caloric formulas were associated with less treatment failure, less weight loss, and shorter LOS compared to lower caloric formula (51). Although low-lactose formulas are perceived to alleviate gastrointestinal issues during the withdrawal period (51), several studies showed that low-lactose formula did not improve NAS outcomes (30, 52, 53).

Although opioid-exposed neonates are born smaller and may have early weight loss, these infants may develop hyperphagia as a compensatory mechanism (54, 55). The growth trajectory of these infants can involve excessive catch-up growth in the first year of life with body composition analysis showing more rapid gain in fat compared to fat-free mass (56, 57). A longitudinal study of cocaine-exposed neonates demonstrated that those born small for gestational age (SGA) developed rapid catch-up growth with a four-fold risk of obesity at nine years of age (58). While this study focused on prenatal cocaine exposure, it would be interesting to examine if opioid-exposed neonates have a similar risk profile. Could the smaller size at birth and abnormal feeding regulation and growth patterns be followed by increased adiposity in childhood and obesity/metabolic syndrome in adulthood? Opioid-exposed neonates may undergo fetal reprogramming (i.e., epigenetic changes) that may contribute to metabolic syndrome, abnormal lipid profiles, and cardiovascular disease in adults with opioid use disorder (59, 60). These studies suggest that opioid-exposed neonates may be at increased risk for nutritional and growth challenges that may persist into adulthood. While physicians are increasingly aware of the need for higher calories and nutritional evaluation for opioid-exposed neonates, there is a great need to advocate for long-term follow-up of infant growth (48, 51).

### Abnormal brain development

Emerging data demonstrate the adverse effects of prenatal opioid exposure on the developing brain at the macrostructural, microstructural, neurophysiological, and/or functional levels. *In utero* opioid exposure results in a smaller head circumference (e.g., altered brain growth), although this effect may be mediated by co-exposure to maternal tobacco or other psychoactive medications (44, 61–64). Early studies using ultrasonography have shown enlargement of in the thalamus of exposed subjects over the first six months of life (65, 66). Amplitude electroencephalographic (aEEG) recordings in opioid-exposed neonates showed increased discontinuity and low voltage recordings, as well as reduced or absent sleep-wake cycling; all these factors were associated with the severity of withdrawal and the need for pharmacotherapy (67–69). aEEG also detected brief seizures in more than half of the infants developing NAS (69).

Magnetic resonance imaging (MRI) has also demonstrated smaller volumes in the basal ganglia, deep gray matter, thalamus, ventrolateral nuclei, brainstem, and cerebrospinal and larger volumes in the right cingulate gyrus and left occipital lobe white matter in NAS (70, 71). Merhar and colleagues reported punctate white matter lesions in the brain of 8 of 20 opioid-exposed neonates (72). In addition to the macrostructural changes, opioid-exposed neonates also have microstructural abnormalities. Diffusion tract imaging of opioid-exposed neonates demonstrated quantitatively and qualitatively reduced fractional anisotropy (FA), which reflects fiber density, axonal diameter, and the degree of myelination, evidence of compromised white matter tract integrity (73, 74). Because reduced FA is associated with motor and cognitive deficits (75), these findings may explain the neurodevelopmental issues experienced by infants with NAS and emphasize the need to monitor this population more closely. The Outcome of Babies with Opioid Exposure (OBOE) study is an ongoing longitudinal cohort study designed to evaluate the impact of prenatal opioid exposure on brain structure-function relationships over the first two years of life (76).

Advanced neuroimaging can provide an even more sophisticated way to demonstrate the adverse impact of prenatal opioid exposure on the developing brain. Radhakrishnan et al. utilized resting-state functional brain MRI and showed significantly higher connectivity between the right amygdala and medial prefrontal region in the exposed cohort (77). Given the role of the amygdala in emotion, stress, and fear and of the prefrontal cortex in the executive function and working memory, this finding has important implications for future addiction-related behavior and risks. Furthermore, alterations in thalamocortical functional connectivity in the brain correlated with the severity of NAS (78). This emphasizes the utility of delineating the subtle yet intricate alterations in neural circuitry caused by prenatal opioid exposure. Another study using resting-state functional MRI also showed that infants with prenatal opioid exposure had smaller network volumes, particularly in the primary visual network, which may explain the higher risk of developmental and visual problems (79).

Visual evoked potentials (VEP) are another method that has demonstrated altered brain functioning in NAS (80). Although VEP does not directly correlate with visual function, it reflects neural maturity and myelination when recording activity over the occipital area. This can provide an objective measure of the visual pathway from the retina to the visual cortex (81). Opioid-exposed neonates have been found to have abnormal VEP including altered morphology, decreased amplitudes, and prolonged peak times (82, 83). These findings either normalized in the first few years of life or persisted until a decade later (80–82, 84), highlighting the importance of ongoing surveillance throughout life in these high-risk infants.

### Neurodevelopmental outcomes and early intervention (EI)

Opioid-exposed neonates are at increased risk for developmental, behavioral, educational, and psychological/mental health issues later in life (85–89). Neonates with NAS requiring pharmacotherapy are even more vulnerable due to *in utero* and postnatal exposures. A multisite, blinded, randomized controlled trial comparing methadone with morphine in NAS demonstrated the superiority of methadone on length of hospital stay, length of stay due to NAS, and length of treatment (90). Despite this finding, a follow-up analysis looking at developmental milestones at 18 months demonstrated that neonates in both treatment arms had similar neurobehavioral deficits and a higher rate of the atypical profile on the NNNS which is associated with worse neurodevelopmental outcomes (91). Furthermore, a higher NAS severity index may be predictive of developmental outcomes at 18 months (92), highlighting the necessity for longitudinal follow-up in these high-risk infants.

Updated AAP guidelines on NAS has emphasized the need for close developmental, behavioral, and mental health screenings after infants are discharged from the hospital (94). All opiod-exposed infants should be referred for comprehensive services (e.g., NICU developmental follow-up programs, EI, etc.) as available. This is a focus of part C of the Individuals with Disabilities Education Act (IDEA) (https://www.cdc.gov/ncbddd/cp/treatment.html) in order to further monitor developmental milestones in these high risk infants (93, 94). Even though EI services are available in all areas in the United States, not all opioid-exposed infants and their families receive these services. Peacock-Chambers et al. showed that in Massachusetts, where the diagnosis of NAS serves as automatic eligibility for one-year EI services, less than half of eligible infants enrolled (95). The rate of EI referral was also shown to vary by custody status (two-fold higher for those discharged with their biological families than foster families) and length of hospital stay (greater referral for those with longer stay). EI referral did not equate to EI enrollment, with only half of referred infants actually enrollment. A national survey also confirmed suboptimal EI referral for opioid-exposed neonates and the discrepancy based on the need for pharmacotherapy, with those requiring pharmacotherapy getting a higher referral rate than those who did not (96). This finding is concerning since all opioid-exposed neonates are at risk for long-term adverse effects, irrespective of the severity of withdrawal and the need for pharmacotherapy (97). Other home-based services, such as the Maternal, Infant, and Early Childhood Home Visiting Program may also benefit these families.

Although a few follow-up studies did not demonstrate significant developmental deficits in children with prenatal opioid exposure, these children can actually demonstrate poorer school performance and worse functioning at adolescence (85, 87). However, these findings may be influenced by food and housing insecurity, psychological and physical stress, and many other environmental factors encountered in childhood. There is an urgent need to study the long-term impact of prenatal opioid exposure which should also include academic and family outcomes to determine if significant differences exist related to the types of treatments (non-pharmacologic/pharmacologic) as well as various therapeutic approaches (scheduled treatments compared to use as needed).

### Ophthalmologic disorders

Neonates with prenatal opioid exposure are at risk for ophthalmologic abnormalities such as strabismus, nystagmus, reduced visual acuity, impaired smooth pursuit, and delayed visual development due to direct neurotoxic effects of opioids and/or other social and neurodevelopmental factors (98–101). A cross-sectional study of children with a history of prenatal opioid exposure showed a 10-fold risk of strabismus in the first three years of life, with the mean age of presentation at 8.3 months (102). Another study showed a 6-fold risk of strabismus and a 90-fold risk of nystagmus in the first five years of life (103). While exodeviations presented earlier in life (6.8 months), esodeviations presented later at 11.6 months (104). A cohort study in a million infants showed that those with NAS had an 8-fold risk of nystagmus, 4.7-fold risk of strabismus, and a 2-fold risk of ophthalmologic-related hospitalization before age 13 (86). A longitudinal cohort study in nearly 800,000 infants showed that substance-exposed infants had a significantly higher incidence of ophthalmologic-related hospital admissions compared to unexposed infants (47.0 vs. 32.0 per 10,000 person-years), with a much higher cumulative incidence that widened over time (399.8 per 10,000 by 12 years of age). Opioids were shown to have a greater impact on ophthalmologic-related hospitalizations than cocaine, cannabis, and others (105). Altogether, evidence supports the association between prenatal opioid exposure, abnormal visuomotor development, and the need for comprehensive anticipatory guidance and timely ophthalmology referrals for this population.

## Conclusion

The study and understanding of NAS has advanced dramatically in the last several decades resulting in tremendous progress in the care of maternal-infant dyads affected by the opioid epidemic. The well-being of these families remains a major public health priority that must look beyond the short-term issues. In addition to efforts to reduce costs and length of hospital stay, clinicians and researchers must provide sound anticipatory guidance that prioritizes multifaceted care surrounding infants with NAS—nutrition, growth, cognitive and neurodevelopmental follow-up, physical therapy, ophthalmologic evaluation, and ample family support. Prenatal opioid exposure is a lifelong process with potentially deleterious effects if not closely monitored. All healthcare, government, industrial, and public health stakeholders must collaborate and advance care that focuses on both the short and longer-term preventive and curative measures for this vulnerable and high-risk population.
